# Variable Effect of HIV Superinfection on Clinical Status: Insights From Mathematical Modeling

**DOI:** 10.3389/fmicb.2018.01634

**Published:** 2018-07-23

**Authors:** Ágnes Móréh, András Szilágyi, István Scheuring, Viktor Müller

**Affiliations:** ^1^MTA Centre for Ecological Research, Danube Research Institute, Budapest, Hungary; ^2^Evolutionary Systems Research Group, MTA Centre for Ecological Research, Tihany, Hungary; ^3^MTA-ELTE Theoretical Biology and Evolutionary Ecology Research Group, Institute of Biology, Eötvös Loránd University, Budapest, Hungary; ^4^Department of Plant Systematics, Ecology and Theoretical Biology, Institute of Biology, Eötvös Loránd University, Budapest, Hungary

**Keywords:** HIV superinfection, AIDS, mathematical model, virus dynamics, invasion analysis

## Abstract

HIV superinfection (infection of an HIV positive individual with another strain of the virus) has been shown to result in a deterioration of clinical status in multiple case studies. However, superinfection with no (or positive) clinical outcome might easily go unnoticed, and the typical effect of superinfection is unknown. We analyzed mathematical models of HIV dynamics to assess the effect of superinfection under various assumptions. We extended the basic model of virus dynamics to explore systematically a set of model variants incorporating various details of HIV infection (homeostatic target cell dynamics, bystander killing, interference competition between viral clones, multiple target cell types, virus-induced activation of target cells). In each model, we identified the conditions for superinfection, and investigated whether and how successful invasion by a second viral strain affects the level of uninfected target cells. In the basic model, and in some of its extensions, the criteria for invasion necessarily entail a decrease in the equilibrium abundance of uninfected target cells. However, we identified three novel scenarios where superinfection can substantially increase the uninfected cell count: (i) if the rate of new infections saturates at high infectious titers (due to interference competition or cell-autonomous innate immunity); or when the invading strain is more efficient at infecting activated target cells, but less efficient at (ii) activating quiescent cells or (iii) inducing bystander killing of these cells. In addition, multiple target cell types also allow for modest increases in the total target cell count. We thus conclude that the effect of HIV superinfection on clinical status might be variable, complicated by factors that are independent of the invasion fitness of the second viral strain.

## 1. Introduction

HIV superinfection occurs when a person already infected with HIV acquires a second (unrelated) strain of the virus. While estimates for the incidence of superinfection vary widely [from virtually zero (Gonzales et al., 2003; Tsui et al., 2004) to rates comparable to that of initial infection (Piantadosi et al., 2008; Redd et al., 2011; Kraft et al., 2012)], the ubiquitous imprint of recombination on the global evolution of HIV diversity (Rambaut et al., 2004; Vuilleumier and Bonhoeffer, 2015) indicates that superinfection cannot be very rare. At the population level, superinfection might affect the evolution of virulence (Nowak and May, 1994; van Baalen and Sabelis, 1995; Alizon and van Baalen, 2008), it might potentially contribute to the spread of drug resistance (Chakraborty et al., 2004; Smith et al., 2005), and, in the case of HIV, it also allows for recombination between distant lineages, which might facilitate adaptation and evolutionary innovation in the virus (Vuilleumier and Bonhoeffer, 2015).

Superinfection can also have an impact on the health status of the affected individual. A number of studies have reported either abrupt deterioration of clinical status (a drop in the CD4+ T cell count and/or increase in the virus load), or accelerated disease progression following superinfection (Altfeld et al., 2002; Jost et al., 2002; Gottlieb et al., 2004, 2007; Yerly et al., 2004; van der Kuyl et al., 2005; Clerc et al., 2010; Cornelissen et al., 2012; Brener et al., 2018). However, there are also counterexamples, where superinfection did not have a negative impact (Casado et al., 2007) or the effect was only transient (Rachinger et al., 2008). Furthermore, superinfection events with no (or, possibly, beneficial) effects might often go unnoticed, as the detection of superinfection requires the sequencing of the viral genome, which is rarely done in unproblematic infections. This led the authors of a comprehensive review on HIV superinfection to conclude that “the full extent and potency of the detrimental effects of superinfection remain unclear and might depend on several viral and host factors” (Redd et al., 2013).

Here, following up on Fung et al. (2010), we use simple mathematical models of HIV infection to analyze a set of biologically relevant scenarios with respect to the possible outcomes of superinfection. Mathematical modeling has been used to study various aspects of the complexity of HIV infection (Nowak and May, 2000; Perelson, 2002; Müller and Bonhoeffer, 2003), including within-host evolution (e.g., Iwasa et al., 2004, 2005) and some scenarios for superinfection (Fung et al., 2010). From an ecological perspective, both cases can be regarded as “invasion tests” (Chesson, 2000): is the second strain (the mutant or the “invader”) able to spread in the steady state (chronic infection) established by the first strain? We use invasion analysis to determine under what conditions a second strain of the virus can establish superinfection, either coexisting with, or excluding the original strain. For the cases where superinfection is successful, we assess the range of possible effects on the uninfected target cell count, which serves as a proxy for the clinical status (health) of the patient. We find that, contrary to intuition, there are biologically plausible scenarios that allow superinfection not only to decrease, but also to increase the target cell count.

## 2. Models and methods

The mathematical framework of virus dynamics describes the interactions between relevant cell and virus types within an infected individual (see e.g., Nowak and May, 2000). Models consist of differential equations that describe the rate of change of each cell and virus type (the variables of the model). We extended the basic model of virus dynamics to explore systematically a set of model variants incorporating various details of HIV infection.

Exposure to superinfection can be implemented by adding a low initial inoculum of a second viral strain to a chronic (steady-state) infection established by the first strain in the models (equivalent to modeling the outcome of within-host mutation events Iwasa et al., 2004). Three outcomes are possible: (i) successful invasion and exclusion of the resident strain; (ii) successful invasion, followed by stable coexistence of both strains; (iii) unsuccessful invasion, the system remains in the original equilibrium with only the resident strain. The invasion is successful (superinfection occurs) if the initial growth rate of the new strain is positive when introduced into the established steady state of the original strain. Exclusion of the original strain occurs if the steady-state cell count of the original strain is zero in the presence of the new strain. Finally, successful invasion results in coexistence if both strains can grow when introduced into a steady-state infection established by the other strain (mutual invasibility).

The impact of superinfection on clinical status can be approximated by comparing the steady-state level of uninfected cells (corresponding to functional CD4+ T cells) before and following the invasion of the superinfecting strain. The possible range of outcomes can be determined by analyzing whether and how the conditions for superinfection constrain the relation of prior and subsequent steady-state target cell levels. In particular, superinfection is strictly associated with the deterioration of clinical status when the (mathematical) conditions for superinfection unambiguously imply that the stable steady-state level of the uninfected cells will be lower in the presence of the invading strain. In this case, only strains that reduce the steady state and thus have negative clinical impact will be able to establish superinfection.

In some of the models, the steady states (equilibrium points) of the system, and the conditions for invasion (and superinfection) could be readily calculated and characterized analytically. In the cases where the analytical approach was impractical due to the complexity of the equations, we employed numerical simulations. We selected credible intervals for all parameter values (Table A5 in Appendix), and then sampled the parameters from their respective intervals independently for each simulation run. We integrated the set of equations corresponding to the uninfected system until equilibrium, then Strain 1 was added. After the system attained steady state (and stable infection with Strain 1 was verified), Strain 2 was added with a low concentration as an invader; the parameters for Strain 2 were selected with the same procedure (including the requirement to establish stable infection given its independently generated set of both viral and host parameters). In case of successful superinfection, we recorded the steady-state level of uninfected target cells both before and after superinfection, along with the corresponding parameter values. We repeated the simulations until we obtained 20000 independent runs with successful superinfection. Numerical integration was performed with the SUNDIALS/CVODE package (Hindmarsh et al., 2005) (C source code is available upon request). In each simulation, we verified the local asymptotic stability of the final steady states by computing the leading eigenvalue of the corresponding Jacobian matrix.

In the following we illustrate the analytical method on a slightly simplified version of the basic model of virus dynamics, then introduce the model variants that we have tested in our analyses.

### 2.1. Basic model

As a starting point, we use a two-strain variant of the established model of virus dynamics, consisting of uninfected target cells (*T*) and two types of infected cells (*I*_1_ and *I*_2_) that harbor the resident and the invading strain of the virus, respectively. The dynamics has the form:

(1) $$\begin{array}{rc} \dot{T} & =\sigma -\left(\beta_{1}I_{1}+\beta_{2}I_{2}\right)T-\delta_{T}T \end{array}$$

(2) $$\begin{array}{rc} \dot{I_{1}} & =\beta_{1}TI_{1}-\delta_{1}I_{1} \end{array}$$

(3) $$\begin{array}{rc} \dot{I_{2}} & =\beta_{2}TI_{2}-\delta_{2}I_{2}, \end{array}$$

where σ is the influx rate, δ_*T*_ is the death rate of uninfected cells, respectively. β_*i*_ denotes the infection efficiency of the *i*th viral strain, and δ_*i*_ is the death rate of cells infected with strain *i*. This is a slightly reduced form of the “basic model of virus dynamics” (Nowak and May, 2000), as it does not explicitly follow the levels of virus particles. This established simplification is justified by the faster turnover of free virions (compared with infected cells), which implies that the concentration of free virions follows (in a quasi steady state) the level of virus producing cells, and the rate of new infections can be made a function of the level of infected cells without loss of generality (Nowak and May, 2000).

The equilibrium values of the target cells can be determined analytically. If infected cells are not present, the system reduces to Equation (1), and the equilibrium value of uninfected cells is ${\hat{T}}^{\left(\right)}=\frac{\sigma }{\delta_{T}}$, where empty brackets in the superscript denote the absence of infection.

If only Strain 1 is present, the corresponding system is Equations (1, 2), and the equilibrium values are: ${\hat{T}}^{\left(I_{1}\right)}=\frac{\delta_{1}}{\beta_{1}}$ and ${Î}_{1}^{\left(I_{1}\right)}=\frac{\sigma }{\delta_{1}}-\frac{\delta_{T}}{\beta_{1}}$. Substituting the uninfected steady state into Equation (2), it follows that infection can be established only if $\frac{\sigma }{\delta_{T}}>\frac{\delta_{1}}{\beta_{1}}$, implying

(4) $$\begin{array}{r} {\hat{T}}^{\left(\right)}>{\hat{T}}^{\left(I_{1}\right)}. \end{array}$$

That is, infection always decreases the uninfected target cell count. Because of the symmetry in the dynamics of infected cells, the same result is obtained for the situation when Strain 2 is present alone. Finally, because $\overset{{}^{\circ}}{I_{1}}=0$ and $\overset{{}^{\circ}}{I_{2}}=0$ are satisfied at different target cell levels (except for the special case when $\frac{\delta_{1}}{\beta_{1}}=\frac{\delta_{2}}{\beta_{2}}$), there is no generic equilibrium point with both strains present. The equilibrium values are listed in Table 1.

**Table 1:** The equilibrium states (*ES*) of the basic model.

	**$\hat{T}$**	**Î_1_**	**Î_2_**
*ES*1 ()	$\frac{\sigma }{\delta_{T}}$	0	0
*ES*2 (*I*_1_)	$\frac{\delta_{1}}{\beta_{1}}$	$\frac{\sigma }{\delta_{1}}-\frac{\delta_{T}}{\beta_{1}}$	0
*ES*3 (*I*_2_)	$\frac{\delta_{2}}{\beta_{2}}$	0	$\frac{\sigma }{\delta_{2}}-\frac{\delta_{T}}{\beta_{2}}$

*The viral strain present in each state is indicated in brackets; empty brackets in ES1 () denote the absence of infection*.

To illustrate the method, in the following we analyze the possibility and the possible outcomes of superinfection in this basic model. The criterion of successful invasion by Strain 2 is the positivity of the growth rate of *I*_2_ (İ_2_>0) in a chronic infection established by the first strain (*ES*2: ${\hat{T}}^{\left(I_{1}\right)}$, ${{Î}_{1}}^{\left(I_{1}\right)}$). By substituting ${\hat{T}}^{\left(I_{1}\right)}$ into Equation (3), it follows that the condition for successful invasion is $\frac{\delta_{1}}{\beta_{1}}>\frac{\delta_{2}}{\beta_{2}}$, which can be rewritten in terms of the equilibrium target cell counts as:

(5) $$\begin{array}{r} {\hat{T}}^{\left(I_{1}\right)}>{\hat{T}}^{\left(I_{2}\right)}, \end{array}$$

implying that successful superinfection always decreases the uninfected target cell count at steady state, because only strains that lower the count can establish superinfection. The criterion for the stable coexistence of both types of infected cells is a positive growth rate of each type of infected cells in the established population of the other. However, mutual invasibility cannot be satisfied as Equation (5) and its reverse cannot be satisfied simultaneously. As a consequence, successful invasion results in the extinction of the resident strain, and the lower steady-state cell count associated with the superinfecting strain is attained.

In this simple system the coexistence of both strains in not possible, and the impact of superinfection is unequivocal. However, implementing some aspects of the complexity of HIV infection can open up the possibility of more complicated behavior in the models. In the following, we introduce extended models of HIV dynamics that incorporate homeostatic target cell dynamics, bystander killing (with or without inducible HIV-specific immunity), interference competition in the infection process, multiple target cell types, or the virus-induced activation of quiescent target cells. The analysis of these models, following the procedure described above, is presented in the Results.

### 2.2. Homeostatic target cell dynamics

The basic model of virus dynamics assumes a constant rate of influx for the susceptible target cells. However, at least some of the new production is likely to arise from the division of existing target cells, and this process must then inevitably be regulated to maintain stable cell counts. Such homeostatic dynamics can be described by a logistic growth term that is a decreasing function of the current size of the cell pool, and we employed the following equation to describe such self-limiting dynamics for the target cells:

(6) $$\begin{array}{r} \dot{T}=rT\left(1-\frac{T}{K}\right)-\left(\beta_{1}I_{1}+\beta_{2}I_{2}\right)T-\delta_{T}T. \end{array}$$

Here *r* defines the maximal per capita growth rate of the uninfected target cells, and *K* is the “carrying capacity” at which divisions stop entirely. Note that we have retained the simple exponential death term (δ_*T*_*T*) for consistence with the basic model, and the dynamics of the infected cells remain unchanged (cf. Equations 2, 3).

### 2.3. Models with bystander killing of uninfected cells

Accumulating evidence indicates that the killing of uninfected cells (induced, primarily, by pyroptosis (Doitsh et al., 2014; Ke et al., 2017) might be a major mechanism of HIV-associated loss of CD4+ T lymphocytes. Viral strains are likely to differ in their ability to induce bystander killing, which gives rise to the following model variant:

(7) $$\begin{array}{rcl} \dot{T} & = & \sigma -\left[\left(\beta_{1}+\gamma_{1}\right)I_{1}+\left(\beta_{2}+\gamma_{2}\right)I_{2}\right]T-\delta_{T}T \end{array}$$

(8) $$\begin{array}{rcl} \dot{I_{1}} & = & \beta_{1}TI_{1}-\delta_{1}I_{1} \end{array}$$

(9) $$\begin{array}{rcl} \dot{I_{2}} & = & \beta_{2}TI_{2}-\delta_{2}I_{2}. \end{array}$$

where the loss of target cells depends not only on the infection efficiency of the strains (β_*i*_, cf. section 2.1), but also on the strength of the bystander killing effect of the infected cells (γ_*i*_).

In addition, inducible immunity that is activated proportional to the level of the antigen can have a profound effect on the equilibria and behavior of the models (De Boer and Perelson, 1998), and indeed on the competition of distinct viral strains (Iwasa et al., 2004). To investigate whether strain-specific immune responses can alter the invasion dynamics of viral strains with varying levels of bystander killing, we combined the earlier model of Iwasa et al. (2004) with bystander killing to obtain the following set of equations:

(10) $$\begin{array}{rcl} \dot{T} & = & \sigma -\underset{i\;=\;1,2}{\sum }\left(\beta_{i}+\gamma_{i}\right)I_{i}T-\delta_{T}T \end{array}$$

(11) $$\begin{array}{rcl} \dot{I_{i}} & = & \beta_{i}TI_{i}-k_{i}E_{i}I_{i}-\delta_{i}I_{i}\;\left(i\;=\;1,2\right) \end{array}$$

(12) $$\begin{array}{rcl} \dot{E_{i}} & = & \alpha_{i}I_{i}E_{i}-\delta_{E_{i}}E_{i}\;\left(i\;=\;1,2\right). \end{array}$$

**Figure 1:**
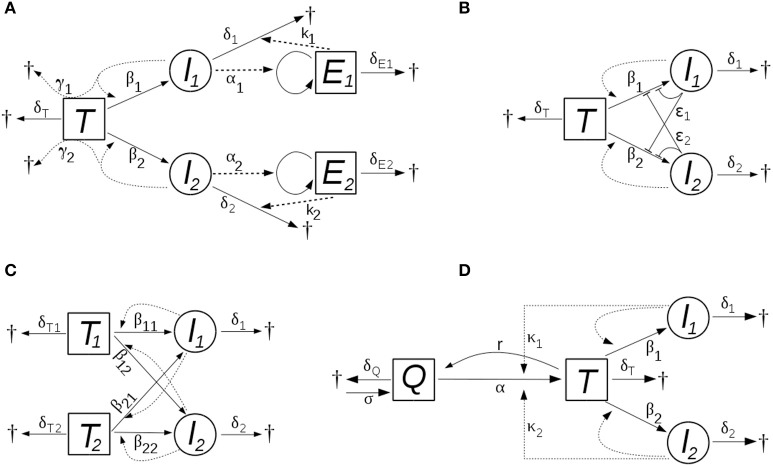
The schemes of the models with **(A)** bystander killing and (optional) strain-specific cytotoxic immunity, **(B)** saturating dynamics of new infections, **(C)** multiple target cell types, and **(D)** HIV induced activation of target cells. New infections occur proportional to the level of infected cells in all models; the level of infectious virions is assumed to follow that of the infected cells, with a proportionality constant implicit in the infection parameter (β). The processes and parameters are explained in the text.

In this model, the two viral strains (i.e., the cells infected by them) activate, and are targeted by, two different populations of effector cells that are specific to the strains. The effector cells proliferate proportional to the level of infected cells with rate constants α_*i*_, die at rates δ_*E*_*i*__, and they kill infected cells in a concentration dependent manner, with rate constants *k*_*i*_. The scheme of the models is shown in Figure 1A.

We also tested model variants with alternative immune effector mechanisms. Cytotoxic lymphocytes might be able to kill newly infected cells before they could start producing virus (Klenerman et al., 1996), which can be implemented by making the fraction of newly infected cells that enter the virus-producing cell population a decreasing function of the immune response:

(13) $$\begin{array}{r} \dot{I_{i}}=\frac{\beta_{i}T}{1+f_{i}E_{\left(i\right)}}I_{i}-\delta_{i}I_{i}\;\left(i\;=\;1,2\right). \end{array}$$

The same equation applies also if some effector cells exert a non-cytotoxic effect that reduces the rate of new infections (Levy et al., 1996); in this case the reduction in the infection terms involves also the loss of uninfected cells:

(14) $$\begin{array}{rc} \dot{T} & =\sigma -\underset{i\;=\;1,2}{\sum }\frac{\beta_{i}I_{i}}{1+f_{i}E_{\left(i\right)}}T-\delta_{T}T \end{array}$$

### 2.4. Saturating dynamics of new infections

Two biological scenarios can be implemented by the following formalism:

(15) $$\begin{array}{rcl} \dot{T} & = & \sigma -\left(\frac{\sum_{i\;=\;1,2}\beta_{i}I_{i}}{1+\sum_{i\;=\;1,2}\epsilon_{i}I_{i}}\right)T-\delta_{T}T \end{array}$$

(16) $$\begin{array}{rcl} \dot{I_{i}} & = & \frac{\beta_{i}TI_{i}}{1+\sum_{i\;=\;1,2}\epsilon_{i}I_{i}}-\delta_{i}I_{i}\;\left(i\;=\;1,2\right), \end{array}$$

in which the rate of new infections increases slower than linearly with increasing infectious titer, and saturates at high titers; ϵ_*i*_ parameters characterize the strength of the effect. First, this can be regarded as a “functional response” in the infection term, acknowledging that the linear proportionality between the rate of infections and the level of infected cells cannot be valid indefinitely as the level of the infected cells increases: at high levels, competitive saturation occurs due to interference (crowding) effects (Schoener, 1978). Alternatively, the same model structure applies also if the presence of the virus induces innate antiviral mechanisms in the target cells (e.g., in the context of abortive infections). HIV is known to be affected by several cell-autonomous innate immune mechanisms (Zheng et al., 2012), some of which are likely to be inducible. In this setting, the effective infection rate might decrease already at lower levels of the infected cells. Figure 1B illustrates the scheme of this model.

### 2.5. Multiple target cell types

Strains of HIV can differ in their target cell tropism, which might also have an effect on their competition dynamics. With regard to the blood CD4+ T cell count (which we use as a proxy for clinical status), the major distinction lies between cells expressing either the CCR5 or the CXCR4 coreceptor (Bleul et al., 1997). Some viral strains are specific for the former, but dual-tropic viruses often evolve during the course of disease progression, with varying levels of affinity for the two coreceptors (Connor et al., 1997). For simplicity, we here investigate two target cell types that are produced independently of each other at rates σ_*i*_, and can be infected by one or both viral strains with coefficients β_*ij*_:

(17) $$\begin{array}{rcl} \dot{T_{i}} & = & \sigma_{i}-T_{i}\underset{j\;=\;1,2}{\sum }\beta_{ij}I_{j}-\delta_{T_{i}}T_{i}\;\left(i\;=\;1,2;j\;=\;1,2\right) \end{array}$$

(18) $$\begin{array}{rcl} \dot{I_{j}} & = & \underset{i\;=\;1,2}{\sum }\beta_{ij}T_{i}I_{j}-\delta_{j}I_{j}\;\left(i\;=\;1,2;j\;=\;1,2\right) \end{array}$$

The total target cell level comprises $\underset{i}{\sum }T_{i}$; the scheme of the model is shown in Figure 1C.

### 2.6. HIV-induced T-cell activation

Our last scenario implements some of the complexity in the dynamics of the target cells of HIV infection. While the majority of CD4+ T cell cells in the body are in a quiescent state, HIV infects only activated cells efficiently (Bukrinsky et al., 1991; Chiu et al., 2005). In addition, the presence of the virus itself might increase the rate of activation, which complicates the dynamics and brings up the possibility that the impact of superinfection might also be affected. Building on earlier models (e.g., Bartha et al., 2008), we consider the following system of equations:

(19) $$\begin{array}{rc} \dot{Q} & =\sigma -\delta_{q}Q-\left(\alpha +\underset{i\;=\;1,2}{\sum }\kappa_{i}I_{i}\right)Q+rT \end{array}$$

(20) $$\begin{array}{rc} \dot{T} & =\left(\alpha +\underset{i\;=\;1,2}{\sum }\kappa_{i}I_{i}\right)Q-\left(r+\delta_{T}\right)T-\underset{i\;=\;1,2}{\sum }\beta_{i}I_{i}T \end{array}$$

(21) $$\begin{array}{rc} {İ}_{i} & =\beta_{i}I_{i}T-\delta_{i}I_{i}\;\left(i\;=\;1,2\right), \end{array}$$

where *T* now denotes activated CD4+ T cells (corresponding, as before, to the susceptible target cells in the system), and *Q* indicates quiescent CD4+ T cells that are in a resting state. Quiescent cells are generated at a constant rate σ, and die at a rate δ_*Q*_*Q*. They become activated at a rate composed of an HIV-independent component, α*Q*, and an HIV-dependent component that is proportional to the level of infected cells, κ_*i*_*I*_*i*_*Q*, where κ_*i*_ denotes the efficiency of activation mediated by the *i*th viral strain. Activated target cells (*T*) revert to quiescent state at the rate *rT*; the death and infection of target cells, and the dynamics of infected cells are the same as in the basic model (see Figure 1D).

Because the dynamics of infected cells is unchanged from the basic model, here, too, coexistence of the two strains is not possible, and successful superinfection always decreases the count of susceptible target cells (*T*). However, in this model the total CD4+ T cell count includes also the quiescent cells, and for this total, the outcome can be different. For details, see section 3.4.

In each scenario we followed the method introduced above, i.e., we investigated the criteria for invasions (mutual invasibility) and the positivity of the steady-state cell levels. We distinguished the possible equilibrium states based on which cell types are present with nonzero steady-state levels at the equilibrium point; we present the distinct equilibrium states of all models in Table 2 for easy reference.

**Table 2:**
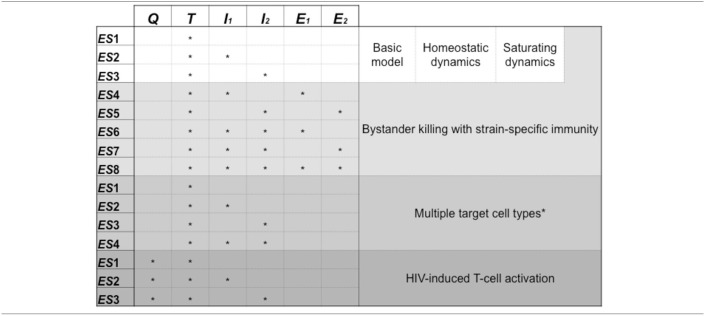
Summary of the possible equilibrium states in the analyzed models, showing the cell types that are present in each equilibrium point.

For analytical forms see Appendix 1–4. Note, that “homeostatic dynamics” refers to the self-limiting dynamics of uninfected target cells, whereas “saturating dynamics” refers to the dynamics of new infections. In the case of multiple target cell types (denoted by ^*^), T refers to the simultaneous presence of both target cell types T_1_andT_2_.

## 3. Results

In Models and Methods we showed that in the basic model of virus dynamics superinfection always entails a decrease in the uninfected target cells. This followed because the criteria for invasion in that model can be fulfilled only for strains that ultimately establish a new steady state of the target cells that is lower than the one set by the resident virus before the invasion. In the following, we use the same methodology of invasion analysis on multiple variants of the HIV dynamics model. The model variants are extensions to the basic model, incorporating various aspects of the complexity of HIV infection. The main results are presented here, while the details of the calculations and simulations are presented in the Appendix. We refer the non-mathematical reader to the beginning of the Discussion, where we summarize the main results in intuitive non-mathematical terms.

### 3.1. Models with uniform negative effect of superinfection

We first briefly discuss the scenarios (model variants) where superinfection either always decreases the uninfected target cell count (as in the basic model), or it might leave the count unchanged in some cases.

#### 3.1.1. Homeostatic target cell dynamics

The equilibrium points of the model are listed in (Table 3). The target cell count in the absence of infection, and the steady states of infected cells differ from those of the basic model of virus dynamics. However, the criteria for successful invasion by a second viral strain, and the steady-state target cell counts before and after superinfection, are derived from the dynamical equations of the infected cells, which are the same as in the basic model. As a consequence, this model variant also predicts a uniform negative impact of superinfection on the target cell level (cf. Equation 5).

**Table 3:** The equilibrium states (*ES*) of the basic model with homeostatic target cell dynamics.

	**$\hat{T}$**	**Î_1_**	**Î_2_**
*ES*1 ()	$\frac{K\left(r-\delta_{T}\right)}{r}$	0	0
*ES*2 (*I*_1_)	$\frac{\delta_{1}}{\beta_{1}}$	$\left({\hat{T}}^{\left(\right)}-{\hat{T}}^{\left(I_{1}\right)}\right)\frac{r}{K\beta_{1}}$	0
*ES*3 (*I*_2_)	$\frac{\delta_{2}}{\beta_{2}}$	0	$\left({\hat{T}}^{\left(\right)}-{\hat{T}}^{\left(I_{2}\right)}\right)\frac{r}{K\beta_{2}}$

We also tested models that combined homeostatic target cell dynamics with other extensions if the basic model, and found that the effect of superinfection was generally independent of the choice between homeostatic dynamics and constant influx of new cells. In the following we therefore present models employing the simpler approximation of constant influx for the uninfected cells, consistent with the basic model.

#### 3.1.2. Bystander killing of uninfected cells

We then studied models that allow for the bystander killing of uninfected cells, which appears to be a major factor in the loss of CD4+ T cells in HIV infection (Doitsh et al., 2014). We aimed to investigate whether differences in the rate of bystander killing can influence the impact of superinfection on clinical status.

Without immune response the dynamics of the system is described by Equations (8–10). The equilibrium points of the system are easily computed (Table 4), revealing that the steady-state counts of uninfected cells remain the same as in the basic model, and only the steady states of the infected cells are different. The relations determining the positivity of the infected cell counts, and the criteria for successful invasion (superinfection) are also unchanged: successful invasion always decreases the uninfected target cell count in this implementation of bystander killing of uninfected target cells.

**Table 4:** Equilibrium states in the case of bystander killing of uninfected cells without immune response.

	**$\hat{T}$**	**Î_1_**	**Î_2_**
*ES*1 ()	$\frac{\sigma }{\delta_{T}}$	0	0
*ES*2 (*I*_1_)	$\frac{\delta_{1}}{\beta_{1}}$	$\frac{\left({\hat{T}}^{\left(\right)}-{\hat{T}}^{\left(I_{1}\right)}\right)\delta_{T}}{{\hat{T}}^{\left(I_{1}\right)}\left(\beta_{1}+\gamma_{1}\right)}$	0
*ES*3 (*I*_2_)	$\frac{\delta_{2}}{\beta_{2}}$	0	$\frac{\left({\hat{T}}^{\left(\right)}-{\hat{T}}^{\left(I_{2}\right)}\right)\delta_{T}}{{\hat{T}}^{\left(I_{2}\right)}\left(\beta_{2}+\gamma_{2}\right)}$

#### 3.1.3. Bystander killing with strain-specific cytotoxic immunity

We next investigated whether an inducible immune response against the virus [which can change the equilibria and behavior of the models profoundly (De Boer and Perelson, 1998)] can affect the outcome of superinfection. Because cross-reactive immunity (that targets both strains) has already been shown to allow for both increasing and decreasing target cell counts after successful invasion (Iwasa et al., 2004), we combined strain-specific immunity with bystander killing. Strain-specific immunity, by itself, does not allow for increasing target cell counts (Iwasa et al., 2004); we aimed to investigate whether immune control by strain-specific immunity might allow for the invasion of a viral strain with reduced bystander killing, possibly increasing the target cell count.

In brief, we found that in models with bystander killing of uninfected cells and strain-specific immunity, superinfection imposed on a steady state with induced immunity always decreases the target cell count (for details see Appendix 1). In the case with an initial virus that is not able to elicit an immune response, superinfection with a fitter virus can result in a situation with stable coexistence, an immune response against the second strain, and no change in the target cell level. Finally, we also tested alternative action mechanisms for the immune response (early cytotoxicity, non-cytotoxic immunity); however, the results of the previous analyses remained robust irrespective of the effector mechanism.

### 3.2. Saturating dynamics of new infections

We next explored whether implementing interference competition between the viral strains can influence the outcome of superinfection. Such competition arises from a “crowding effect” that reduces the per capita rate of new infections at high virus load, acknowledging that the rate of new infections cannot increase indefinitely with the level of infected cells. Alternatively, the same model applies also if innate antiviral mechanisms are activated in the target cells proportional to the virus load they are exposed to.

In this model variant there is no immune control and infected cell originate from a single pool of target cells (see Figure 1B); the coexistence of both strains is therefore not possible. The dynamics of the system is described in Equations (16, 17), where the rate of new infections increases slower than linearly with increasing infectious titer, and saturates at high titers. The three possible equilibrium points are listed in Table A2 in Appendix 2.1. In the case of successful superinfection the new strain excludes the old one. The condition of successful invasion by the second strain has the same form as in the basic model (for details, see Appendix 2.2):

(22) $$\begin{array}{r} \frac{\delta_{1}}{\delta_{2}}>\frac{\beta_{1}}{\beta_{2}}. \end{array}$$

However, in this model, the total target cell count can both decrease and increase after successful superinfection. The count increases if the following relation holds:

(23) $$\begin{array}{r} \left(\delta_{1}\beta_{2}-\delta_{2}\beta_{1}\right)+\delta_{T}\left(\delta_{1}\epsilon_{2}-\delta_{2}\epsilon_{1}\right)+\sigma \left(\epsilon_{1}\beta_{2}-\epsilon_{2}\beta_{1}\right)<0. \end{array}$$

As the expression in the first pair of brackets must be positive for superinfection to occur (c.f. Equation 23), the relation can hold if the sum of the remaining two expressions is negative and of greater magnitude. If σ≫δ_*T*_ (which is a realistic assumption) the condition is mainly affected by the ϵ_*i*_ coefficients of interference and the β_*i*_ coefficients of infection efficiency, yielding the following necessary (though not sufficient) condition for an increase in the target cell count after superinfection:

(24) $$\begin{array}{r} \frac{\epsilon_{1}}{\epsilon_{2}}<\frac{\beta_{1}}{\beta_{2}}. \end{array}$$

If σ≪δ_*T*_ the condition is mainly affected by the δ_*i*_ rates of infected cell turnover, in addition to the coefficients of interference, and an increase in the target cell count is possible only if

(25) $$\begin{array}{r} \frac{\epsilon_{1}}{\epsilon_{2}}<\frac{\delta_{1}}{\delta_{2}}. \end{array}$$

In general, superinfection can increase the level of uninfected target cells, if the relative difference between the two strains is smaller with respect to the coefficients of interference than with respect to the relative difference in the infection efficiency and/or in the infected cell turnover. As interference by a “crowding effect” is likely to be relatively invariable, this condition might often be fulfilled under this scenario.

**Figure 2:**
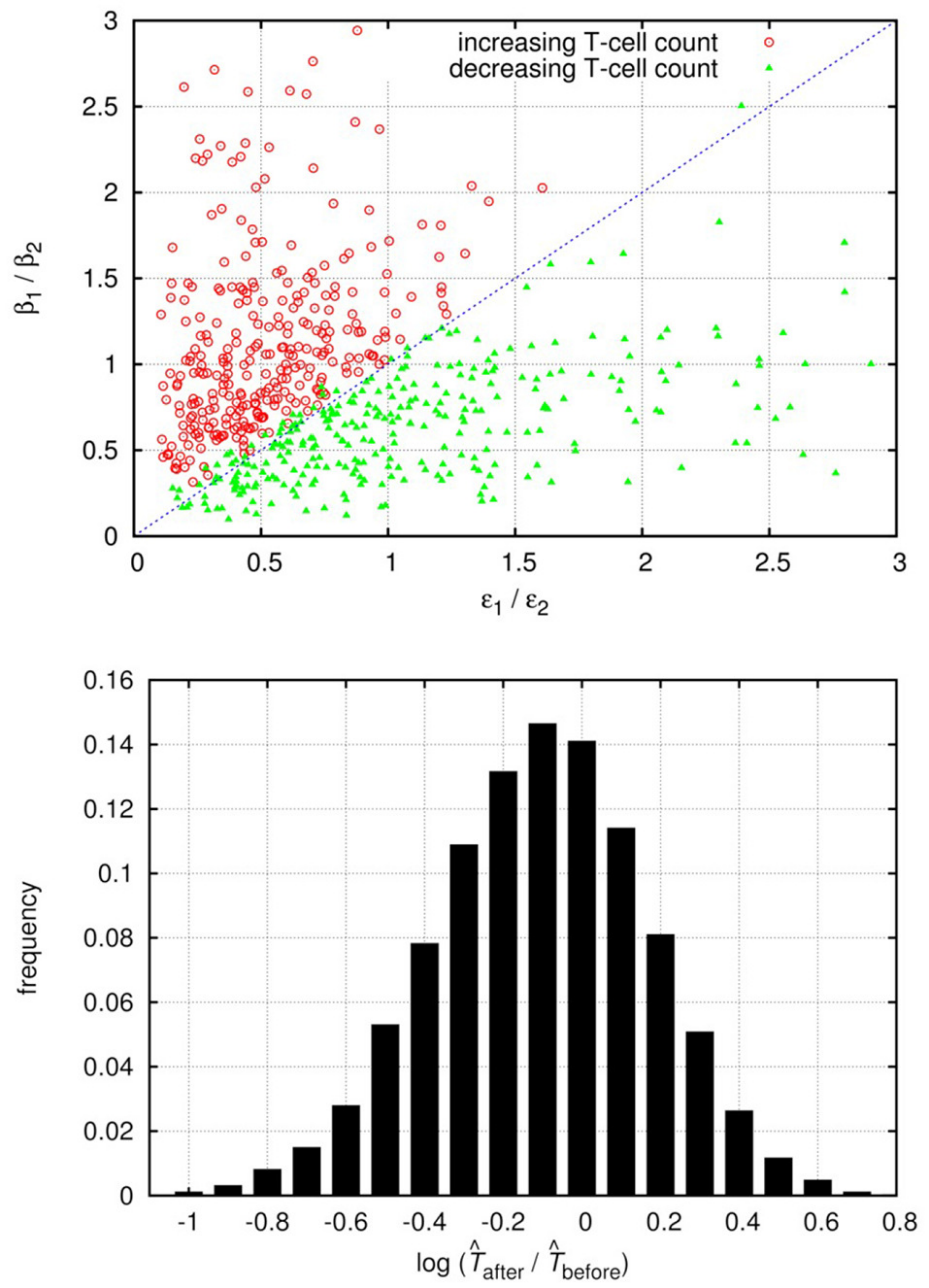
The top panel shows the change in the uninfected target cell count after superinfection as a function of the relative differences in the interference (ϵ) and infection efficiency (β) parameters of both strains; results from 600 randomly selected simulation runs of the saturating infection dynamics model (300–300 runs with both increasing and decreasing cell counts) are shown. Red circles represent runs with increasing uninfected target cell count; green triangles represent runs with decreasing cell counts. The blue dashed line of the diagonal corresponds to $\frac{\epsilon_{1}}{\epsilon_{2}}=\frac{\beta_{1}}{\beta_{2}}$; Equation (25) is fulfilled above the diagonal. In all runs we set σ = 10 cells per day and δ_*T*_ = 0.1 per day; all other parameters were drawn randomly with uniform distribution from the intervals presented in Table A5 (Appendix). The lower panel shows the histogram of the (log-transformed) ratios of the uninfected target cell counts after and before superinfection, from 20,000 simulation runs with successful superinfection.

As the above calculations are only approximate, we also carried out a series of numerical simulations to investigate the effect of superinfection on the uninfected target cell count. We fixed the parameters of the uninfected cells such that σ≫δ_*T*_, when the condition for increasing target cell count is expected to be approximated by $\frac{\epsilon_{1}}{\epsilon_{2}}<\frac{\beta_{1}}{\beta_{2}}$; all other parameters were chosen randomly from the intervals presented in Table A5 in Appendix. Overall about 50% of the invasion tests resulted in successful superinfection (from a random pair of strains, one can always exclude the other, except for the degenerate case when β_1_/δ_1_ = β_2_/δ_2_). In each run the increase/decrease of the uninfected target cell counts after the superinfection and the ratios of β_*i*_ and ϵ_*i*_ parameters were recorded. Figure 2 shows the results from a randomly selected subset of simulations with successful superinfection (300 cases of both increasing and decreasing target cell counts), confirming the validity of the approximate criterion; the distribution of the relative change in the cell count is shown for the whole set of 20,000 simulation runs with successful superinfection.

### 3.3. Multiple target cell types

This model variant was motivated by the observation that different virus strains can differ in their target cell tropism (e.g., Bleul et al., 1997), which might influence their competition dynamics by introducing multiple resources into the system. The scheme of the model is shown in Figure 1C. With two target cell types, exposure to a second strain can lead to three different outcomes: unsuccessful invasion; successful superinfection with exclusion of the original strain; and successful superinfection followed by the coexistence of both strains. There are four equilibrium states of the system, but the complexity of their form (c.f. Appendix 3.1) precludes an analytical investigation of the effect of superinfection. We therefore assessed the impact of superinfection with numerical simulations of the model, using parameters sampled randomly from credible intervals (see Table A5 in Appendix), and recording the total number of target cells (*T*_1_+*T*_2_) before and after a successful superinfection (see Models and Methods for details). The ratio of simulations with successful superinfection was again, as expected, close to 50%. In 20,000 simulation runs with successful superinfection, the most frequent scenario was the exclusion of the first strain accompanied by a decrease in the total uninfected target cell count (*T*_1_+*T*_2_); however, a modest increase in the total count was also observed in some of the cases (Table 5), and coexistence of the two strains was also possible with both increasing and decreasing total uninfected target cell counts. We found no parameters or simple parameter combinations that could predict the increase or decrease of total counts.

**Table 5:** The observed frequencies of the possible outcomes of successful superinfection, and the median and interquartile range of the ratio of change in the uninfected target cell count for each case, calculated from 20,000 simulation runs with successful superinfection (50% of the total number of runs) in the multiple target cell types model.

**Outcome**	**Frequency**	**Median ratio**
		**of change (Q1–Q3)**
Exclusion–increasing total count	0.005	1.029 (1.010−1.062)
Exclusion–decreasing total count	0.815	0.467 (0.290−0.672)
Coexistence–increasing total count	0.020	1.010 (1.003−1.033)
Coexistence–decreasing total count	0.160	0.889 (0.759−0.965)

### 3.4. HIV-induced activation of target cells

Our final extension of the basic model takes into account that only activated CD4+ T cells are highly susceptible to HIV infection, while the majority of the CD4+ T cells are in a resting or quiescent state. By equating the susceptible target cells (*T*) with activated T cells, the model can preserve much of the basic architecture, while adding a new variable for the levels of quiescent cells (*Q*) allows it to track the total CD4+ T cell count with more realism. An important feature of the system is that HIV itself contributes to the activation of quiescent cells. The dynamics of the system is described by the set of differential equations introduced in Equations (20–22); the scheme of the model is shown in Figure 1D. The three equilibrium states (*ES*1, *ES*2, and *ES*3; see Table 2, but note that *Q* is also present) and the corresponding equilibrium values of different cell counts can be found in Appendix 4.1.

As there is no immune control, and both strains of the virus infect the same pool of (activated) target cells, coexistence of strains is not possible, analogous to the basic model (cf. section 2.1). In the case of successful invasion, the original strain is excluded, and the level of activated target cells decreases, in line with the results of the basic model: $\hat{T}\left(I2\right)<\hat{T}\left(I1\right)$, see Equation (5). In the equilibrium states with infection, the steady-state values of susceptible target cell levels, *T*, are the same in the basic model and this model; however, the addition of quiescent cells allows for a more complicated behavior of the total uninfected target cell count (*Q*+*T*) in this case. From Equation (20), the steady-state level of quiescent cells can be expressed in the following way:

(26) $$\begin{array}{r} {\hat{Q}}^{\left(I_{i}\right)}=\frac{r{\hat{T}}^{\left(I_{i}\right)}+\sigma }{\delta_{q}+\alpha +\kappa_{i}{Î}^{\left(I_{i}\right)}}. \end{array}$$

While the complexity of the fully expanded formula of the steady state (see Appendix 4.1) precludes a fully analytical study of the possible consequences of superinfection, the possibility of increasing cell count can be gleaned by expressing the increase of the total CD4+ T cell count (${\hat{Q}}^{\left(I_{2}\right)}+{\hat{T}}^{\left(I_{2}\right)}>{\hat{Q}}^{\left(I_{1}\right)}+{\hat{T}}^{\left(I_{1}\right)}$) in the following form:

(27) $$\begin{array}{r} \frac{\delta_{2}}{\beta_{2}}+\frac{r\frac{\delta_{2}}{\beta_{2}}+\sigma }{\delta_{q}+\alpha +\kappa_{2}{Î}^{\left(I_{2}\right)}}>\frac{\delta_{1}}{\beta_{1}}+\frac{r\frac{\delta_{1}}{\beta_{1}}+\sigma }{\delta_{q}+\alpha +\kappa_{1}{Î}^{\left(I_{1}\right)}}. \end{array}$$

**Figure 3:**
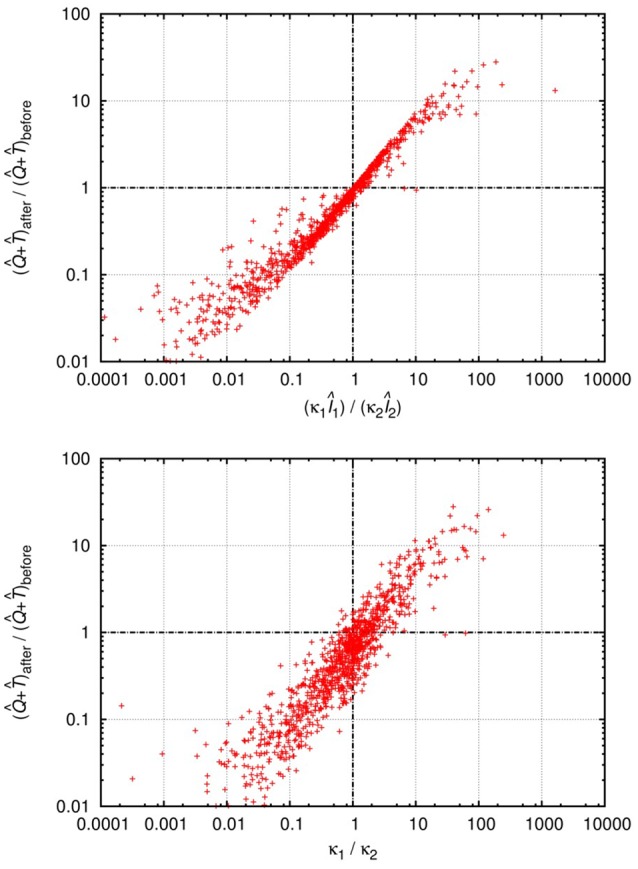
Relative change of the total uninfected target cell count ($\hat{Q}+\hat{T}$) after and before successful superinfection, plotted against the total rates of activation (κ_1_Î_1_)/(κ_2_Î_2_) (top) or the ratio of the activation parameters κ_1_/κ_2_ (bottom) of the two virus strains in the HIV-induced activation model. The results of 20,000 simulation runs with successful superinfection are shown. In each run, all parameters were drawn randomly with uniform distribution from the intervals presented in Table A5 (Appendix); the cases with healthy (uninfected) cell counts between 500 and 1,500 per μL were used for the analyses. Both axes are logarithmic.

Although the level of activated target cells decreases, (i.e., δ_2_/β_2_ < δ_1_/β_1_), the inequality can be fulfilled if the invading Strain 2 exerts a (sufficiently) lower level of virus-mediated target cell activation (κ_2_Î_2_ < κ_1_Î_1_), which might be possible for some parameter combinations. We tested this by numerical integration of the set of differential equations Equations (20–22), following the method used in the previous two scenarios (for details see section 2). In about 10% of the cases, with single infection the system attained stable oscillations with large amplitude in all variables, which is biologically unrealistic; we have therefore excluded these cases from further analysis. We performed invasion tests with pairs of strains that both attained stable equilibria in single infections; of these tests, about 11% resulted in successful superinfection. This is considerably lower than the “neutral” expectation observed in the other models, and can be explained by the additional positive feedback of infected cell levels on the supply of susceptible (activated) cells. The second strain still has 50% probability to have higher replicative fitness (β/δ) than the resident strain; however, in some of these cases it has too low activation potential to sustain infection in the new host. The results of 20,000 successful invasions are presented in Figure 3.

In line with the qualitative predictions, the total target cell count increased for some cases of superinfection where the (κ_1_*I*_1_)/(κ_2_*I*_2_) ratio was greater than 1. The κ_1_/κ_2_ ratio was also a good proxy: substantial increase in the total target cell count seems to be possible only if κ_1_/κ_2_>1, i.e., when the invading strain is less efficient at activating quiescent target cells. Based on these numerical results, we conclude that the total uninfected cell count can both decrease and increase after superinfection, if the dynamics of target cell activation and quiescence is taken into account.

Finally, we also tested a minor variant of this model, in which quiescent cells affected by the virus die instead of entering the pool of activated target cells [i.e., the κ_*i*_*I*_*i*_ terms appear only in the equation of quiescent cells (Equation 20) but not in the equation of activated cells (Equation 21)]. This formalism corresponds to a mechanism of bystander killing that affects resting uninfected cells, which might apply to the pyroptotic pathway in particular (Doitsh et al., 2014). The behavior of this model was analogous to the structurally similar case of HIV-induced T cell activation: superinfection with a strain that has higher replicative capacity but a lower rate of HIV-induced bystander killing of the quiescent cells, compared with the resident strain, can increase the total CD4+ T cell count.

## 4. Discussion

Using simple models of HIV infections, we demonstrated that superinfection with a second strain of HIV can, under different assumptions, result in both a deterioration, but also an improvement of clinical status (approximated by uninfected target cell counts in the models). This runs counter to the widespread view that associates superinfection with a negative outcome. In our exploration of biologically motivated extensions to the basic model of HIV dynamics, we have identified four new scenarios in which superinfection can also have a positive impact on the level of uninfected target cells.

The first scenario assumed interference competition for the susceptible target cells between the competing viral clones. Such interference is almost inevitable at high densities of a predator or infectious agent (Schoener, 1978): the rate of new infections cannot grow indefinitely with increasing infectious titer. Furthermore, the same model structure is applicable also if inducible mechanisms of innate antiviral defense reduce the susceptibility of uninfected cells upon exposure to the virus that does not result in productive infection. Interference competition (saturating infection dynamics) can therefore be expected to occur, although the magnitude of the effect is unclear. In this model, the total uninfected target cell count increased upon superinfection when the relative difference between the two viral strains was smaller with respect to the coefficients of interference than with respect to the relative difference in the infection efficiency and/or in the infected cell turnover.

Variable tropism for multiple distinct cell types also allowed for increasing total uninfected cell counts, although in this case the increase was modest and it occurred in only a minority of the simulation runs with randomized parameters. Larger increases in the total count were possible in models that distinguished between activated (susceptible) and resting (non-permissive) target cells, and included an effect of the virus on the resting cell pool (activation to susceptible state or bystander killing). In these models, “invasion fitness” of a virus strain is independent of its effect on quiescent cells, allowing for superinfection with strains that induce less depletion of this cell pool, which constitutes the dominant component of the total CD4+ T cell count.

In all scenarios that allow for increasing target cell level after superinfection, this positive outcome is expected to arise (in some of the cases) when there are independent sources of variability in the relevant parameters, e.g., if the intensity of interference effects, or the potential for immune activation can vary, at least in part, independent of the components of replicative fitness (production and infectiousness of virions, turnover rates of infected cells and virus particles). Since a complete coupling is not expected between the parameters, the possibility of increasing target cell levels is likely if any of the relevant structural features of these scenarios is indeed important *in vivo*. This is a robust result, independent of the uncertainties in the parameters of both viral and host immune dynamics.

Our results add to the earlier modeling work of Fung et al. who found that HIV superinfection can occur with a less fit (and virulent) strain if target cells can be multiply infected (which reduces or eliminates competition for this resource) (Fung et al., 2010). Furthermore, since exposure to superinfection is fully analogous to the appearance of new virus strains by mutation, earlier modeling results pertaining to the within-host emergence and competition of new strains are also applicable in the context of superinfection (e.g., Iwasa et al., 2004, 2005; Ball et al., 2007), and vice versa. Altogether, there are now five mechanisms known to allow for a positive impact of HIV superinfection on clinical status (uninfected target cell count): in addition to the four cases identified in this paper, the earlier work of Iwasa et al. (2004) identified cross-reactive immunity as a mechanism that is also compatible with a positive outcome – all of these scenarios could, in principle, also allow for evolution toward decreasing HIV virulence within the host. We summarize the predictions of various mathematical models with regard to the impact of HIV superinfection on clinical status in Table 6.

**Table 6:** Possible outcomes of HIV superinfection on the total uninfected target cell count.

**Scenario**	**After superinfection**	**Source**
	**the target cell count**
Basic model	Always decreases	Iwasa et al., 2004
Homeostatic target cell dynamics	Always decreases	This paper
Strain-specific immunity	Decreases or unchanged	Iwasa et al., 2004
Cross-specific immunity	Can decrease or increase	Iwasa et al., 2004, 2005
Multiple infection of target cells	Decreases or unchanged^*^	Fung et al., 2010
Bystander killing		
*of susceptible target cells*	Always decreases	This paper
*of non-permissive target cells*	Can decrease or increase	This paper
Saturating infection dynamics	Can decrease or increase	This paper
Multiple target cell types	Can decrease or increase	This paper
HIV-induced T-cell activation	Can decrease or increase	This paper

* *Fung et al. (2010) used a non-steady-state model of disease progression: when dual infection of the target cells was allowed to occur unhindered, the rate of disease progression was unaffected or slightly accelerated after superinfection*.

While modeling suggests that HIV superinfection could have counterintuitive beneficial effects by several possible mechanisms, the data are not sufficient to predict how often this might occur. Elucidating the true distribution of outcomes might be elusive in the era of broadly accessible antiretroviral therapy, but it might be possible through the retrospective identification of superinfection events from stored samples. Finding cases where the CD4+ T cell count improved, at least temporarily, after superinfection, would indicate that at least one of the complicating factors that allow such an effect are indeed at work in the infection. Insights from the models and a detailed examination of these cases could narrow down the list of possible mechanisms, and improve our understanding of the within-host dynamics of HIV infection.

Finally, our results might also have some relevance with regard to the impact of superinfection on the evolution of HIV virulence at the population level. The possibility of ambiguous outcomes implies that superinfection might contribute to the spreading of not only virulent, but also of attenuated strains under some circumstances. We also note that even in the scenarios when superinfection could spread only strains with higher virulence, this predicted effect could be mitigated by factors that were not incorporated in our models. For example, the initial dissemination of the virus is likely to be aided considerably by the large susceptible population of CD4+CCR5+ T cells in the gut-associated lymphoid tissue (Mehandru et al., 2004). This pool is quickly and irreversibly depleted when an individual first becomes infected with HIV, and the absence of this readily infectable cell population might reduce the probability of successful superinfection upon subsequent exposure to other viral strains. This and other factors (e.g., cross-specific immunity) might inhibit superinfection, which would constrain the spreading of strains with higher within-host fitness also at the population level (Ferdinandy et al., 2015). Furthermore, the current broad application of antiretroviral therapy is likely to reduce also the incidence of superinfection, especially considering that therapeutic guidelines increasingly advise the treatment of all diagnosed individuals. In principle, superinfection by drug resistant viruses could still occur (Chakraborty et al., 2004; Smith et al., 2005), but currently available evidence suggests that such events are extremely rare (Bartha et al., 2013). Finally, the population-level dynamics and evolution of HIV is also influenced by factors that act on between-host transmission (Nowak and May, 1994; van Baalen and Sabelis, 1995; Alizon and van Baalen, 2008), and trade-offs between viral traits might also complicate the evolutionary dynamics (Ball et al., 2007).

In summary, we have shown that the effect of HIV superinfection on clinical status is not straightforward: while the simplest models predict that only a more virulent strain can successfully establish superinfection, adding biologically relevant details of HIV infection opens up the possibility that superinfection might also improve clinical status in some cases. The impact of superinfection at the population (epidemic) level is likely to be modulated by further factors.

## Author contributions

VM conceived and supervised the study. ÁM, AS, IS, and VM developed the models. ÁM, AS, IS, and VM performed the analyses. ÁM, AS, IS, and VM wrote the paper.

### Conflict of interest statement

The authors declare that the research was conducted in the absence of any commercial or financial relationships that could be construed as a potential conflict of interest.
