# Supplementary material for: Variable Effect of HIV Superinfection on Clinical Status: Insights From Mathematical Modeling
Source: Front Microbiol. 2018 Jul 23;9:1634. doi: 10.3389/fmicb.2018.01634 (PMC6064737; doi:10.3389/fmicb.2018.01634)
Supplement: Supplementary file 1 [file Data_Sheet_1.pdf]

Appendix to *Variable effect of HIV superinfection on clinical status: insights from mathematical modeling*

Ágnes Móréh [1], **András Szilágyi** [2,3], **István Scheuring** [2,3] **and Viktor Müller** [2,4*]

[1] *Danube Research Institute, MTA Centre for Ecological Research, Budapest, Hungary*
[2] *Evolutionary Systems Research Group, MTA Centre for Ecological Research, Tihany, Hungary*
[3] *MTA-ELTE Theoretical Biology and Evolutionary Ecology Research Group, Institute of Biology, Eötvös Loránd University, Budapest, Hungary*
[4] *Department of Plant Systematics, Ecology and Theoretical Biology, Institute of Biology, Eötvös Loránd University, Budapest, Hungary*

Correspondence*:
Viktor Müller
mueller.viktor@ttk.elte.hu

In the Appendix we present the derivation of some results in more detail. The steady-state cell levels of the equilibrium states of each model are listed in tables. The variables for infected and effector cells that take non-zero values in a given equilibrium are denoted in brackets in superscript. As target cells are always present, we do not denote their presence separately (we denote the uninfected equilibrium with empty brackets).

Each section corresponds to a given extension of the basic model; the methodology of the analysis is the same as outlined in the main text. (i) We assume that the infected system is initially in $ES4$, (ii) we check conditions for the growth of the invader strain in the established $ES4$, then (iii) we check the invasion ability of the old strain into the established population of a new strain (mutual invasibility), and (iv) the positivity of all variables in the respective states. From these we can characterize the conditions for invasibility, coexistence or competitive exclusion, and the corresponding change in the target cell counts.

1 BYSTANDER KILLING WITH STRAIN SPECIFIC IMMUNITY

In this scenario each virus strain activates, and is affected by, a different population of effector cells. This system follows the dynamics described by Eqs. (10-12), and has eight equilibrium points, listed in Table A1.

	$\hat{T}$	$\hat{I}_1$	$\hat{I}_2$	$\hat{E}_1$	$\hat{E}_2$
$ES1\,()$	$\frac{\sigma}{\delta_T}$	0	0	0	0
$ES2\,(I_1)$	$\frac{\delta_1}{\beta_1}$	$\frac{(\hat{T}^{()}-\hat{T}^{(I_1)})\delta_T}{\hat{T}^{(I_1)}(\beta_1+\gamma_1)}$	0	0	0
$ES3\,(I_2)$	$\frac{\delta_2}{\beta_2}$	0	$\frac{(\hat{T}^{()}-\hat{T}^{(I_2)})\delta_T}{\hat{T}^{(I_2)}(\beta_2+\gamma_2)}$	0	0
$ES4\,(I_1;E_1)$	$\frac{\sigma}{\delta_T+(\beta_1+\gamma_1)\frac{\delta_{E1}}{\alpha_1}}$	$\frac{\delta_{E1}}{\alpha_1}$	0	$\frac{\beta_1}{k_1}(\hat{T}^{(I_1;E_1)}-\hat{T}^{(I_1)})$	0
$ES5\,(I_2;E_2)$	$\frac{\sigma}{\delta_T+(\beta_2+\gamma_2)\frac{\delta_{E2}}{\alpha_2}}$	0	$\frac{\delta_{E2}}{\alpha_2}$	0	$\frac{\beta_2}{k_2}(\hat{T}^{(I_2;E_2)}-\hat{T}^{(I_2)})$
$ES6\,(I_{1,2};E_1)$	$\hat{T}^{(I_2)}$	$\frac{\delta_{E1}}{\alpha_1}$	$\frac{(\hat{T}^{(I_1,E_1)}-\hat{T}^{(I_{1,2},E_1)})\sigma}{\hat{T}^{(I_1,E_1)}\hat{T}^{(I_{1,2},E_1)}(\beta_2+\gamma_2)}$	$\frac{\beta_1}{k_1}(\hat{T}^{(I_{1,2};E_1)}-\hat{T}^{(I_{1,2};E_2)})$	0
$ES7\,(I_{1,2};E_2)$	$\hat{T}^{(I_1)}$	$\frac{(\hat{T}^{(I_2,E_2)}-\hat{T}^{(I_{1,2},E_2)})\sigma}{\hat{T}^{(I_2,E_2)}\hat{T}^{(I_{1,2},E_2)}(\beta_1+\gamma_1)}$	$\frac{\delta_{E2}}{\alpha_2}$	0	$\frac{\beta_2}{k_2}(\hat{T}^{(I_{1,2};E_2)}-\hat{T}^{(I_{1,2};E_1)})$
$ES8\,(I_{1,2};E_{1,2})$	$\frac{\sigma}{\delta_T+(\beta_1+\gamma_1)\frac{\delta_{E1}}{\alpha_1}+(\beta_2+\gamma_2)\frac{\delta_{E2}}{\alpha_2}}$	$\frac{\delta_{E1}}{\alpha_1}$	$\frac{\delta_{E2}}{\alpha_2}$	$\frac{\beta_1}{k_1}(\hat{T}^{(I_{1,2};E_{1,2})}-\hat{T}^{(I_{1,2};E_2)})$	$\frac{\beta_2}{k_2}(\hat{T}^{(I_{1,2};E_{1,2})}-\hat{T}^{(I_{1,2};E_1)})$

Table A1 Equilibrium states of the model with bystander killing and strain-specific cytotoxic immunity.

As a starting point of the invasion tests (corresponding to exposure to superinfection), we assume that the resident strain (I_1) and the effector cells specific to it (E_1) are present initially, i.e., the system starts in the $ES4$ state. Note that the target cell counts are identical between the states $ES3$ and $ES6$, and also between states $ES2$ and $ES7$ ($\hat{T}^{(I_{1,2};E_1)} = \hat{T}^{(I_2)}$ and $\hat{T}^{(I_{1,2};E_2)} = \hat{T}^{(I_1)}$). If the invader strain does not elicit an immune response after superinfection, then the target cell count settles to the level controlled by the invader strain, even if the original strain survives in stable coexistence.

Exposure to the invader strain can have three types of outcomes: (1) coexistence with the resident strain ($ES6$, $ES7$, $ES8$, depending on the presence/absence of either or both immune responses); (2) exclusion of the resident strain ($ES5$, $ES3$) and (3) unsuccessful invasion ($ES4$ remains). In this scenario, we need to investigate the criteria of not only the invasion of the second strain, but also of the activation of an immune response specific to it. The possible outcomes are summarized in Fig. A1.

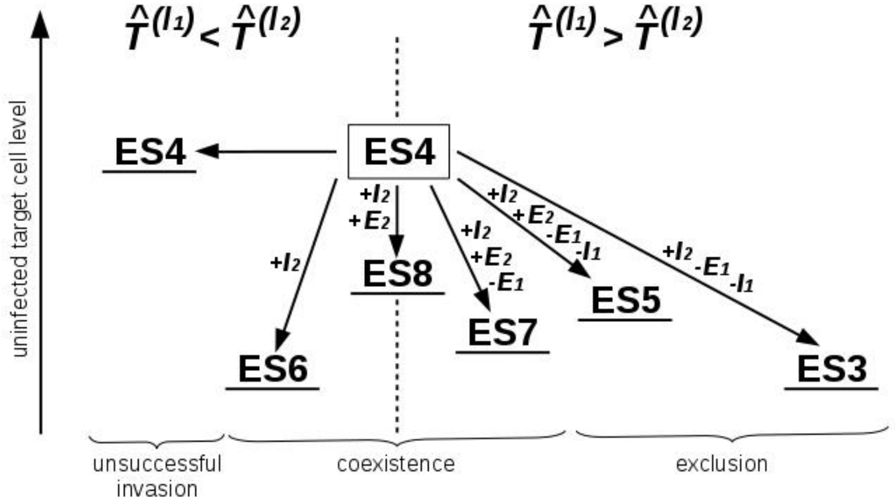

Figure A1. Possible outcomes of exposure to a second viral strain in the model with strain-specific immunity. $ES4$ is the initial state; the relative vertical position of the equilibrium states indicates (in a qualitative manner) the relations between the steady-state target cell levels of the states. Letters on arrows indicate the entry or loss of cell types.

The criteria for invasion and immune activation

In the initial state ($ES4$) the target cells infected by the resident strain (I_1) and the effector cells (E_1) activated by these infected cells are present. As shown earlier, the target cell count is higher in the presence than in the absence of an immune response, i.e. $\hat{T}^{(I_1,E_1)} > \hat{T}^{(I_1)}$.
The condition for the growth of the second strain ($\dot{I}_2 > 0$) in an infection established by the first strain ($ES4$) is the following:

$$\beta_2 \hat{T}^{(I_1,E_1)} - \delta_2 > 0 \tag{A1}$$

$$\hat{T}^{(I_1,E_1)} > \frac{\delta_2}{\beta_2} \tag{A2}$$

$$\hat{T}^{(I_1,E_1)} > \hat{T}^{(I_2)} \tag{A3}$$

Since $\hat{T}^{(I_2)} = \hat{T}^{(I_{1,2},E_1)}$, it follows that

$$\hat{T}^{(I_1,E_1)} > \hat{T}^{(I_{1,2},E_1)}. \tag{A4}$$

Thus, successful superinfection without the activation immunity against the superinfecting strain ($ES4 \rightarrow ES6$ or $ES3$) decreases the uninfected target cell count. The same result can be obtained by investigating the steady-state I_2 cell count, since $\hat{I}_2^{(I_{1,2},E_1)}$ is positive, if $\hat{T}^{(I_1,E_1)} > \hat{T}^{(I_{1,2},E_1)}$. In addition, $\hat{E}_1^{(I_{1,2},E_1)}$ is positive, if

$$\hat{T}^{(I_{1,2},E_1)} > \hat{T}^{(I_{1,2},E_2)} \tag{A5}$$

$$\hat{T}^{(I_2)} > \hat{T}^{(I_1)} \tag{A6}$$

$$\vdots$$

$$\frac{\beta_1}{\beta_2} > \frac{\delta_1}{\delta_2}. \tag{A7}$$

The condition for the growth of E_2 ($\dot{E}_2 > 0$) in $ES6$ is the following:

After a successful invasion, the second strain can activate effector cells against itself ($\dot{E}_2 > 0$), i.e., the $ES6 \rightarrow ES8$ shift can occur if

$$\alpha_2 \hat{I}_2^{(I_{1,2},E_1)} - \delta_{E_2} > 0 \tag{A8}$$

$$\frac{(\hat{T}^{(I_1,E_1)} - \hat{T}^{(I_{1,2},E_1)})\sigma}{\hat{T}^{(I_1,E_1)}\hat{T}^{(I_{1,2},E_1)}(\beta_2 + \gamma_2)} > \frac{\delta_{E_2}}{\alpha_2} \tag{A9}$$

After a short rearrangement we obtain

$$\frac{(\hat{T}^{(I_1,E_1)} - \hat{T}^{(I_{1,2},E_1)})}{\hat{T}^{(I_1,E_1)}\hat{T}^{(I_{1,2},E_1)}} > \frac{\frac{\delta_{E2}}{\alpha_2}(\beta_2 + \gamma_2)}{\sigma} \tag{A10}$$

$$\vdots$$

$$\frac{1}{\hat{T}^{(I_{1,2},E_1)}} > \frac{1}{\hat{T}^{(I_{1,2},E_{1,2})}} \tag{A11}$$

$$\hat{T}^{(I_{1,2},E_{1,2})} > \hat{T}^{(I_{1,2},E_1)} \tag{A12}$$

(Note that, we can arrive at the same results by investigating the steady-state E_2 cell count, since $\hat{E}_2^{(I_{1,2},E_{1,2})}$ is positive, if $\hat{T}^{(I_{1,2},E_{1,2})} > \hat{T}^{(I_{1,2},E_1)}$.)

For coexistence of both strains, the first strain also has to be able to grow when introduced into a chronic infection by the invading strain. The condition for the growth of I_1 in $ES5$ (note that here $\hat{T}^{(I_2,E_2)} > \hat{T}^{(I_2)}$) is the following:

$$\beta_1\hat{T}^{(I_2,E_2)} - \delta_1 > 0 \tag{A13}$$

$$\hat{T}^{(I_2,E_2)} > \frac{\delta_1}{\beta_1} \tag{A14}$$

$$\hat{T}^{(I_2,E_1)} > \hat{T}^{(I_1)} \tag{A15}$$

Since $\hat{T}^{(I_1)} = \hat{T}^{(I_{1,2},E_2)}$, it follows that

$$\hat{T}^{(I_2,E_2)} > \hat{T}^{(I_{1,2},E_2)}. \tag{A16}$$

Thus, successful superinfection – without immune activation – decreases further the uninfected target cell count also in case of the other strain. (Note that the same results can be obtained by investigating the steady-state I_1 cell count, since $\hat{I}_1^{(I_{1,2},E_2)}$ is positive, if $\hat{T}^{(I_2,E_2)} > \hat{T}^{(I_{1,2},E_2)}$.) Next, it can be shown that $\hat{E}_2^{(I_{1,2},E_2)}$ is positive if

$$\hat{T}^{(I_{1,2},E_2)} > \hat{T}^{(I_{1,2},E_1)} \tag{A17}$$

$$\hat{T}^{(I_1)} > \hat{T}^{(I_2)} \tag{A18}$$

$$\vdots$$

$$\frac{\beta_1}{\beta_2} < \frac{\delta_1}{\delta_2}. \tag{A19}$$

Further, E_1 is able to grow in $ES7$ if

$$\alpha_1\hat{I}_1^{(I_{1,2},E_2)} - \delta_E > 0. \tag{A20}$$

Following the same process of rearrangements as in the case of the $ES6 \to ES8$ shift, we obtain

$$\hat{T}^{(I_{1,2},E_{1,2})} > \hat{T}^{(I_{1,2},E_2)}. \tag{A21}$$

(The same condition can be derived by the equilibrium E_1 cell count, since $\hat{E}_1^{(I_{1,2},E_{1,2})}$ is positive if $\hat{T}^{(I_{1,2},E_{1,2})} > \hat{T}^{(I_{1,2},E_2)}$.)

In this case coexistence of both strains can occur in several equilibrium states that differ in the presence or absence of the strain-specific immune responses. Fig. A1 shows the possible routes from $ES4$ to different final states. Note that not all states can be reached directly from $ES4$.

As it is presented above, not all of the criteria can be realized at the same time. While the inequalities Eqs. (A4) and (A12), or Eqs. (A16) and (A21) can be true at the same time, Eqs. (A6) and (A18) exclude each other. This means that coexistence is possible, but it can be reached by different paths depending on the relation between the parameters β_i and δ_i.

The possible outcomes of superinfection

The possible outcomes can be grouped according to the relative replication capacity (expressed by $\frac{\beta_i}{\delta_i}$) of the original and the invader strain.

1. The original strain is more infectious ($\frac{\beta_1}{\beta_2} > \frac{\delta_1}{\delta_2}$).
   In this case, without immune control the original strain sets a lower equilibrium target cell count, and (as in the basic model) superinfection would be unsuccessful in the absence of effector cells. With immunity, the invader strain can either coexist with the resident strain ($ES6$ or $ES8$), or it cannot establish superinfection ($ES4$ remains).
   - Coexistence without immune activation ($ES4 \to ES6$)
     The second strain can spread if it decreases the equilibrium target cell count below the level set by the resident strain with immune response ($\hat{T}^{(I_1;E_1)} > \hat{T}^{(I_{1,2};E_1)} (= \hat{T}^{(I_2)})$). Thus, successful invasion without immune activation occurs, if

     $$\hat{T}^{(I_1)} < \hat{T}^{(I_2)} < \hat{T}^{(I_1;E_1)}. \tag{A22}$$

     As long as the second strain does not elicit an immune response, the system is in the $ES6$ state. (Note that switching the roles of the two strains in this scenario would result in an $ES2 \to ES7$ transition, from an initial infection with no immunity, to stable coexistence after superinfection with a fitter virus strain that elicits an immune response, and no change in the target cell level).
   - Coexistence with immune activation ($ES4 \to ES6 \to ES8$)
     As it is shown in Eq. (A12), the activation of the immune response results in a higher target cell count ($\hat{T}^{(I_{1,2};E_{1,2})} > \hat{T}^{(I_{1,2};E_1)}$). Comparing the equilibrium target cell counts in $ES4$ and $ES8$ we obtain $\hat{T}^{(I_1,E_1)} > \hat{T}^{(I_{1,2},E_{1,2})}$, since

     $$\frac{\hat{T}^{(I_1,E_1)}}{\hat{T}^{(I_{1,2},E_{1,2})}} = \frac{\delta_T + (\beta_1 + \gamma_1)\frac{\delta_{E_1}}{\alpha_1} + (\beta_2 + \gamma_2)\frac{\delta_{E_2}}{\alpha_2}}{\delta_T + (\beta_1 + \gamma_1)\frac{\delta_{E_1}}{\alpha_1}}. \tag{A23}$$

     Thus, the following relations between the target cell counts need to be fulfilled:

     $$\hat{T}^{(I_1)} < \underbrace{\hat{T}^{(I_{1,2},E_1)}}_{\hat{T}^{(I_2)}} < \hat{T}^{(I_{1,2},E_{1,2})} < \hat{T}^{(I_1,E_1)}. \tag{A24}$$

For successful invasion of a new strain with lower infection efficiency, it is necessary that the immune response – specific to the resident strain – would increase the target cell count to a level that is sufficient for the invader to spread. Even stronger immune pressure on the initial strain can allow the second strain to grow to a level where it activates an immune response against itself.

- Unsuccessful invasion ($ES4$ remains)
This occurs when $\dot{I}_2 < 0$ in $ES4$, which requires:

$$\hat{T}^{(I_1)} < \hat{T}^{(I_1,E_1)} < \underbrace{\hat{T}^{(I_{1,2},E_1)}}_{\hat{T}^{(I_2)}}. \tag{A25}$$

In this case, immune pressure on the first strain is too weak to compensate for the disadvantage of the second strain with respect to replicative capacity.

2. The invader strain is more infectious ($\frac{\beta_1}{\beta_2} < \frac{\delta_1}{\delta_2}$).
In this case the invader strain can always spread in the established population of the resident. In the absence of immunity – as the invader strain is viable at lower target cell level – the original strain would be excluded. Inducible immunity allows the possibility of coexistence in the system.

- Coexistence with immune activation ($ES4 \to ES8$)
Similar to the previous cases, it can be shown that the condition for this transition is: $\hat{T}^{(I_{1,2};E_{1,2})} > \hat{T}^{(I_{1,2};E_2)}$. Considering that $\hat{T}^{(I_{1,2};E_2)} = \hat{T}^{(I_1)}$, we can write:

$$\hat{T}^{(I_2)} < \hat{T}^{(I_1)} < \hat{T}^{(I_{1,2},E_{1,2})} < \hat{T}^{(I_1,E_1)}. \tag{A26}$$

The possibility of this case again depends on a strong immune control of the fitter virus variant (Strain 2 in this case), such that the first strain could not only survive, but could also maintain its immune response.

- Coexistence with immune activation, and deactivation of the immune control of the resident strain ($ES4 \to ES7$)
If the new strain decreases the target cell count below the critical level at which the immune response against the resident strain could be maintained (i.e., $\dot{E}_1 < 0$ at $ES7$), then the original immune response is lost after superinfection. The conditions for this case can be written as:

$$\hat{T}^{(I_2)} < \hat{T}^{(I_{1,2},E_{1,2})} < \underbrace{\hat{T}^{(I_1)}}_{\hat{T}^{(I_{1,2},E_2)}} < \hat{T}^{(I_1,E_1)}. \tag{A27}$$

As long as these relations are true, coexistence is possible between the two strains. However, superinfection with a strain of higher replicative capacity can also result in the exclusion of the original strain, after which the new strain may or may not elicit a new immune response.

- Exclusion of the resident strain with immune activation ($ES4 \to ES5$)
Exclusion occurs if $\hat{T}^{(I_2,E_2)} < \hat{T}^{(I_{1,2},E_2)}$ (c.f. Eq. (A16)). Considering that $\hat{T}^{(I_1,E_1)} > \hat{T}^{(I_1)}$ also holds, we can write the following condition for this case:

$$\hat{T}^{(I_2)} < \hat{T}^{(I_2,E_2)} < \hat{T}^{(I_1)} < \hat{T}^{(I_1,E_1)}, \tag{A28}$$

implying that the exclusion of the initial strain (and superinfection) is accompanied by a decrease in the uninfected target cell count.

- Exclusion of the resident strain without immune activation ($ES4 \to ES3$)
  It can also occur that although the second strain excludes the original one, it is not able to elicit a new immune response. For this case we obtain the following conditions:

$$\hat{T}^{(I_2,E_2)} < \hat{T}^{(I_2)} < \hat{T}^{(I_1)} < \hat{T}^{(I_1,E_1)}. \tag{A29}$$

In summary, in these models with bystander killing of uninfected cells and strain-specific immunity, superinfection imposed on a steady state with induced immunity always decreases the target cell count. In the case with an initial virus that is not able to elicit an immune response, superinfection with a fitter virus can result in a situation with stable coexistence, an immune response against the second strain, and no change in the target cell level. Finally, we also tested alternative action mechanisms for the immune response (early cytotoxicity, non-cytotoxic immunity); however, the results of the previous analyses remained robust irrespective of the effector mechanism.

2 SATURATING DYNAMICS OF NEW INFECTIONS

2.1 Equilibrium states

	$\hat{T}$	$\hat{I}_1$	$\hat{I}_2$
$ES1$ ()	$\frac{\sigma}{\delta_T}$	0	0
$ES2$ (I_1)	$\frac{\delta_1+\epsilon_1\sigma}{\beta_1+\epsilon_1\delta_T}$	$\frac{\beta_1\sigma-\delta_1\delta_T}{\delta_1(\beta_1+\epsilon_1\delta_T)}$	0
$ES3$ (I_2)	$\frac{\delta_2+\epsilon_2\sigma}{\beta_2+\epsilon_2\delta_T}$	0	$\frac{\beta_2\sigma-\delta_2\delta_T}{\delta_2(\beta_2+\epsilon_2\delta_T)}$

Table A2 Equilibrium states of the model with saturating dynamics of new infections

2.2 The criteria for invasion and for the possible outcomes of superinfection

The second strain can grow ($\dot{I}_2 > 0$) in the established infection of the first strain ($ES2$) if

$$\beta_2\hat{T}^{(I_1)} - \delta_2 - \delta_2\epsilon_1\hat{I}^{(I_1)} > 0. \tag{A30}$$

By substituting $\hat{T}^{(I_1)}$ and $\hat{I}^{(I_1)}$, after some rearrangement we get the following condition for successful superinfection:

$$\frac{\delta_1}{\delta_2} > \frac{\beta_1}{\beta_2}. \tag{A31}$$

As coexistence is not possible, successful superinfection entails the exclusion of the original strain, and the new equilibrium is characterised by $\hat{T}^{(I_2)}$. The steady-state count of uninfected target cells increases if

$$\hat{T}^{(I_2)} > \hat{T}^{(I_1)} \tag{A32}$$

$$\frac{\delta_2 + \epsilon_2\sigma}{\beta_2 + \epsilon_2\delta_T} > \frac{\delta_1 + \epsilon_1\sigma}{\beta_1 + \epsilon_1\delta_T} \tag{A33}$$

$$\vdots$$

$$(\delta_1\beta_2 - \delta_2\beta_1) + \delta_T(\delta_1\epsilon_2 - \delta_2\epsilon_1) + \sigma(\epsilon_1\beta_2 - \epsilon_2\beta_1) < 0 \tag{A34}$$

3 MULTIPLE TARGET CELL TYPES

3.1 Equilibrium states

	$\hat{T}_1$	$\hat{T}_2$	$\hat{I}_1$	$\hat{I}_2$
$ES1$ ()	$\frac{\sigma}{\delta_{T_1}}$	$\frac{\sigma}{\delta_{T_1}}$	0	0
$ES2$ (I_1)	#	#	#	0
$ES3$ (I_2)	#	#	0	#
$ES4$ ($I_1; I_2$)	$\frac{\beta_{22}\delta_1-\beta_{21}\delta_2}{\beta_{11}\beta_{22}-\beta_{12}\beta_{21}}$	$\frac{\beta_{11}\delta_2-\beta_{12}\delta_1}{\beta_{11}\beta_{22}-\beta_{12}\beta_{21}}$	$\frac{\beta_{12}\delta_{T_2}-\beta_{22}\delta_{T_1}}{\beta_{11}\beta_{22}-\beta_{12}\beta_{21}}+ +\frac{\beta_{22}\sigma_1}{\beta_{22}\delta_1-\beta_{21}\delta_2}+\frac{\beta_{12}\sigma_2}{\beta_{12}\delta_1-\beta_{11}\delta_2}$	$\frac{\beta_{21}\delta_{T_1}-\beta_{11}\delta_{T_2}}{\beta_{11}\beta_{22}-\beta_{12}\beta_{21}}+ +\frac{\beta_{21}\sigma_1}{\beta_{21}\delta_2-\beta_{22}\delta_1}+\frac{\beta_{11}\sigma_2}{\beta_{11}\delta_2-\beta_{12}\delta_1}$

Table A3 Equilibrium states of the model with two target cell types. Some steady-state levels are too complicated to be presented for any practical use; these are denoted with #.

4 HIV-INDUCED T CELL ACTIVATION

4.1 Equilibrium states

	$\hat{Q}$	$\hat{T}$	$\hat{I}_1$	$\hat{I}_2$
$ES1$ ()	$\frac{(r+\delta_T)\sigma}{r\delta_q+\alpha\delta_T+\delta_q\delta_T}$	$\frac{\alpha\sigma}{r\delta_q+\alpha\delta_T+\delta_q\delta_T}$	0	0
$ES2$ (I_1)	$\frac{A\pm\sqrt{-4\beta_1\delta_q\kappa_1(r\delta_1^2+\beta_1\delta_1\sigma)+(-A)^2}}{2\beta_1\delta_q\kappa_1}$	$\frac{\delta_1}{\beta_1}$	$\frac{\sigma-\hat{Q}^{(I_1)}(\delta_q+\alpha)+r\hat{T}^{(I_1)}}{\hat{Q}^{(I_1)}\kappa_1}$	0
$ES3$ (I_2)	$\frac{B\pm\sqrt{-4\beta_2\delta_q\kappa_2(r\delta_2^2+\beta_2\delta_2\sigma)+(-B)^2}}{2\beta_2\delta_q\kappa_2}$	$\frac{\delta_2}{\beta_2}$	0	$\frac{\sigma-\hat{Q}^{(I_2)}(\delta_q+\alpha)+r\hat{T}^{(I_2)}}{\hat{Q}^{(I_2)}\kappa_2}$

Table A4 Equilibrium states of the model with HIV-induced T cell activation.

$A = \alpha\beta_1\delta_1 + \beta_1\delta_1\delta_q - \delta_1\delta_T\kappa_1 + \beta_1\kappa_1\sigma$
$B = \alpha\beta_2\delta_2 + \beta_2\delta_2\delta_q - \delta_2\delta_T\kappa_2 + \beta_2\kappa_2\sigma$

4.2 The total uninfected target cell count before and after superinfection

$$\dot{Q}^{(I_i)} = 0 \tag{A35}$$

$$\sigma + r\hat{T}^{(I_i)} - (\delta_q + \alpha + \kappa_i\hat{I}_i)\hat{Q}^{(I_i)} = 0 \tag{A36}$$

$$\vdots$$

$$\hat{Q}^{(I_i)} = \frac{r\hat{T}^{(I_i)} + \sigma}{\delta_q + \alpha + \kappa_i\hat{I}_i} \tag{A37}$$

The total uninfected target cell count increases after superinfection if

$$\hat{Q}^{(I_1)} + \hat{T}^{(I_1)} < \hat{Q}^{(I_2)} + \hat{T}^{(I_2)} \tag{A38}$$

$$\frac{r\frac{\delta_1}{\beta_1} + \sigma}{\delta_q + \alpha + \kappa_1 \hat{I}_1} + \frac{\delta_1}{\beta_1} < \frac{r\frac{\delta_2}{\beta_2} + \sigma}{\delta_q + \alpha + \kappa_2 \hat{I}_2} + \frac{\delta_2}{\beta_2} \tag{A39}$$

$$\frac{\delta_1}{\beta_1} - \frac{\delta_2}{\beta_2} < \frac{r\frac{\delta_2}{\beta_2} + \sigma}{\delta_q + \alpha + \kappa_2 \hat{I}_2} - \frac{r\frac{\delta_1}{\beta_1} + \sigma}{\delta_q + \alpha + \kappa_1 \hat{I}_1}. \tag{A40}$$

As the left side of the inequality is positive, it follows that

$$\frac{r\frac{\delta_2}{\beta_2} + \sigma}{\delta_q + \alpha + \kappa_2 \hat{I}_2} > \frac{r\frac{\delta_1}{\beta_1} + \sigma}{\delta_q + \alpha + \kappa_1 \hat{I}_1} \tag{A41}$$

$$\frac{\delta_q + \alpha + \kappa_1 \hat{I}_1}{\delta_q + \alpha + \kappa_2 \hat{I}_2} > \frac{r\frac{\delta_1}{\beta_1} + \sigma}{r\frac{\delta_2}{\beta_2} + \sigma} \tag{A42}$$

$$\vdots$$

$$\kappa_1 \hat{I}_1 > \kappa_2 \hat{I}_2. \tag{A43}$$

5 PARAMETERS FOR NUMERICAL SIMULATIONS

The estimation of parameters for HIV dynamics is notoriously difficult for both viral (Müller et al., 2001; De Boer et al., 2010) and lymphocyte (Borghans and De Boer, 2007; De Boer and Perelson, 2013) parameters – not due to a lack of data, but due to the uncertainties in the structure of the processes involved. Wherever possible, we used independent estimates for the parameters, instead of adopting estimates that fitted all parameters of the basic model of virus dynamics simultaneously (e.g., Luo et al. (2012)), as the latter relied on the assumption that changes in the total blood CD4+ T cell count and plasma RNA load after the start of treatment is representative of the relevant processes, which is likely to be confounded by multiple factors. For example, HIV readily infects activated dividing CD4+ T cells only (Bukrinsky et al., 1991), which comprise one to a few percent of the total CD4+ T cell pool (Hellerstein et al., 1999; Hazenberg et al., 2003), amounting to about 10 cells/μL in the blood. These cells are lost from the suceptible pool mostly due to reversion to resting state, which can occur with a rate as fast as 1/day (Fleury et al., 1998), implying $\delta_T \leq$ 1/day, and an input of up to 10 cells/μL/day (consistent with Hellerstein et al. (1999)) to maintain the observed uninfected steady state. We varied both parameters in a broad range, to reflect the uncertainty not only in parameters but even in the identity of the relevant cell populations. The turnover rate of infected cells is relatively reliably estimated after perturbation of the chronically infected steady state by antiretroviral treatment, in the range of 0.1/day $< \delta_I <$ 1/day (Perelson et al., 1996; Markowitz et al., 2003). We then varied the infection parameter β in a broad range, allowing for efficient (median $\sim$50% depletion of the susceptible target cell pool. The activation rate of quiescent T cells (α) was set to reflect the low frequency of activated (dividing) cells; their death rates were selected from a broad range to reflect the considerable variation in the published estimates and also the heterogeneity (naive vs. memory cells) of this pool (Borghans and De Boer, 2007). Because broad variation in all dynamic parameters of the uninfected cells collectively resulted in very broad ranges for the steady-state cell counts in the HIV-induced target cell activation model, we then filtered the random parameter sets of this model to restrict the healthy

steady-state count of uninfected cells between 500 and 1500 cells per μL. The parameter of HIV-induced target cell activation (κ) was set such that the total rate of activation would increase several-fold compared with the uninfected steady state, to reflect empirical data (Hellerstein et al., 1999; Hazenberg et al., 2003). Finally, the strength of the saturation effect in the infection dynamics is unknown; we set the parameter ϵ such that the interference effect could vary substantially in the typical range of the infected cell count.

Overall, we need to emphasize that the extensive uncertainties in the parameters imply that the numerical results presented here should be viewed as 'semi-quantitative'. However, we note also that in the relevant model variants the possibility of increasing target cell counts after superinfection depends only on the presence of variation in the parameters among viral strains, and not on the exact ranges of these parameters. The ranges used in our simulations are summarized in Table A5.

Parameter	Range	Description (units)	References
$\sigma_{(i)}$	$1 - 10$	influx of new target cells (c d^{-1})	Hellerstein et al. (1999) and calculated
$\delta_{T_{(i)}}$	$0.1 - 1$	turnover rate of uninfected target cells (d^{-1})	Fleury et al. (1998)
δ_i	$0.1 - 1$	turnover rate of infected cells (d^{-1})	Perelson et al. (1996), Markowitz et al. (2003)
$\beta_{i(j)}$	$0.01 - 0.2$	infection efficiency (c^{-1} d^{-1})	calculated
δ_Q	$10^{-4} - 10^{-3}$	death rate of quiescent target cells (d^{-1})	Borghans and De Boer (2007)
α_i	$0.002 - 0.02$	natural activation rate of quiescent target cells (d^{-1})	calculated
r	$0.1 - 1$	rate of reversion to quiescent state (d^{-1})	Fleury et al. (1998)
κ_i	$0 - 0.02$	HIV-induced activation rate (c^{-1} d^{-1})	calculated
ϵ_i	$0.1 - 1$	strength of saturating effect (c^{-1})	calculated

Table A5 Parameters used in the numerical simulations of the models. Indices indicate that parameters might vary depending on the viral strain and/or target cell type. For each simulation run, parameters were drawn independently with uniform probability from the ranges provided. Abbreviations: c=cell(s)/μL; d=day.

REFERENCES

Borghans, J. A. M. and De Boer, R. J. (2007). Quantification of T-cell dynamics: from telomeres to DNA labeling. *Immunological Reviews* 216, 35–47. doi:10.1111/j.1600-065X.2007.00497.x

Bukrinsky, M., Stanwick, T., Dempsey, M., and Stevenson, M. (1991). Quiescent T lymphocytes as an inducible virus reservoir in HIV-1 infection. *Science* 254, 423–427. doi:10.1126/science.1925601

De Boer, R. J. and Perelson, A. S. (2013). Quantifying T lymphocyte turnover. *Journal of Theoretical Biology* 327, 45 – 87. doi:https://doi.org/10.1016/j.jtbi.2012.12.025

De Boer, R. J., Ribeiro, R. M., and Perelson, A. S. (2010). Current estimates for HIV-1 production imply rapid viral clearance in lymphoid tissues. *PLOS Computational Biology* 6, 1–9. doi:10.1371/journal.pcbi.1000906

Fleury, S., de Boer, R. J., Rizzardi, G. P., Wolthers, K. C., Otto, S. A., Welbon, C. C., et al. (1998). Limited CD4+ T-cell renewal in early HIV-1 infection: effect of highly active antiretroviral therapy. *Nat Med* 4, 794–801

Hazenberg, M. D., Otto, S. A., van Benthem, B. H., Roos, M. T., Coutinho, R. A., Lange, J. M., et al. (2003). Persistent immune activation in HIV-1 infection is associated with progression to AIDS. *AIDS* 17, 1881–8. doi:10.1097/01.aids.0000076311.76477.6e

Hellerstein, M., Hanley, M. B., Cesar, D., Siler, S., Papageorgopoulos, C., Wieder, E., et al. (1999). Directly measured kinetics of circulating T lymphocytes in normal and HIV-1-infected humans. *Nature Medicine* 5, 83. doi:10.1038/4772

Luo, R., Piovoso, M. J., Martinez-Picado, J., and Zurakowski, R. (2012). HIV model parameter estimates from interruption trial data including drug efficacy and reservoir dynamics. *PLoS One* 7, e40198. doi:10.1371/journal.pone.0040198

Markowitz, M., Louie, M., Hurley, A., Sun, E., Di Mascio, M., Perelson, A. S., et al. (2003). A novel antiviral intervention results in more accurate assessment of human immunodeficiency virus type 1 replication dynamics and T-cell decay in vivo. *J Virol* 77, 5037–8

Müller, V., Marée, A. F. M., and De Boer, R. J. (2001). Release of virus from lymphoid tissue affects human immunodeficiency virus type 1 and hepatitis C virus kinetics in the blood. *Journal of Virology* 75, 2597–2603. doi:10.1128/JVI.75.6.2597-2603.2001

Perelson, A. S., Neumann, A. U., Markowitz, M., Leonard, J. M., and Ho, D. D. (1996). HIV-1 dynamics in vivo: virion clearance rate, infected cell life-span, and viral generation time. *Science* 271, 1582–6
